# What happens after forensic psychiatric care? A latent class analysis of dimensions of welfare for former forensic psychiatric patients

**DOI:** 10.1186/s12888-023-05428-x

**Published:** 2023-12-12

**Authors:** Ebba Noland, Fia Klötz Logan, Stefan Sjöström, Mattias Strandh

**Affiliations:** 1 Department of Social Work, Umeå University, Umeå, 901 87 Sweden; 2 Sundsvall Forensic Psychiatric Centre, Region Västernorrland, Box 880, Sundsvall, 851 24 Sweden; 3 Department of Sociology, Center for social work, Uppsala University, Box 624, Uppsala, Sweden

**Keywords:** Forensic psychiatry, Mentally disordered offender, Criminal recidivism, Latent class analysis, Level of living, Welfare dimensions, Life situation, Post-discharge

## Abstract

**Background:**

Mentally disordered offenders are a heterogenous group regarding psychopathology as well as background factors, which makes it likely that more than one stereotypical life situation will apply to all forensic psychiatric patients following discharge. Knowledge about typical life situations would be valuable for optimising support for improving the overall life situation of these individuals. This paper investigates life situations from the perspective of level of living research and resources in terms of different welfare dimensions.

**Methods:**

Included were all all individuals (*n* = 1146) who had been discharged from forensic psychiatric care in Sweden during 2009–2018 and were included in the Swedish National Forensic Psychiatric Register. Follow-up time varied from 4 to 3644 days, (*m* = 1697, *Md* = 1685). Register data from several different registers was combined. Data was analysed using latent class analysis, and multinominal logistic regression analysis investigated what background factors were associated with class membership.

**Results:**

The results show that there are four subgroups of post-discharge life situations: the high support group, the general psychiatric needs group, the working group, and the family group. The high support group was the largest, representing 54% of the entire sample. There are background factors associated with group membership, including both age at discharge, length of stay in forensic psychiatric care and pre-index crime historical factors.

**Conclusions:**

This study contributes to the understanding of the post-discharge lives of former forensic psychiatric patients and shows that for several subgroups, negative outcomes are rare. Knowledge about these subgroups could be drawn upon to make informed decisions about in- and outpatient forensic psychiatric care, discharge from forensic psychiatric services, and what support is offered to former forensic psychiatric patients.

**Supplementary Information:**

The online version contains supplementary material available at 10.1186/s12888-023-05428-x.

## Background

Mentally disordered offenders (MDOs) is a legally (rather than medically) defined group, and they are a heterogenous group when it comes to psychopathology, criminal history, and risk factors for reoffending [1]. However, there is very little research about the post-discharge lives of this group. The heterogeneity of the group also makes it likely that there is more than one stereotypical life situation that will apply to all MDOs. In this study, the group is referred to as former forensic psychiatric patients (FFPs), to be more precise.

There are considerable knowledge gaps regarding forensic psychiatric patients overall [2] but some areas are more extensively researched than others. Research on this group has focused on psychiatric diagnoses, previously-committed offenses [1, 3], and adverse outcomes such as recidivism [4–6] and mortality [7]. A perspective that is missing from both existing literature on FFPs and other offenders is a wider view of offenders’ complete life situation. Knowing more about the life situations of FFPs has intrinsic value and is also important because knowledge about typical life situations would enable optimisation of support types and interventions given. This, in turn, could help to improve the overall life situation of these individuals.

### Welfare research

Investigating the ‘life situation’ of an individual can be done in a multitude of ways, using a number of different terminologies. One way of doing this is the ‘level of living’ approach. In the tradition of welfare research, level of living is a key concept (see, for example, S Johansson [8]). “Welfare” has been used as an umbrella term for an array of dimensions that are ultimately influenced collectively by political decisions, and the different components have been defined in terms of “resources”.

In level of living research, social and economic indicators are used to move from a strictly material concept of welfare to a wider concept that includes universal social concerns as well as economic aspects [9]. This concept organises different components mainly into the sectors used in social policy and has been defined as an “individual’s command over resources in terms of money, possessions, health, education, family, social and civic rights, etc.” [9]. This perspective is based on a theoretical assumption that resources help individuals shape their own lives [10]. Level of living research is therefore meaningful even though it can only measure the ‘objective’ frame of welfare that is expressed in figures and statistics without taking experiences of welfare into consideration [11, 12]. Level of living research has been criticised for lacking any potential for causal analysis as opposed to mere description. However, it has also been concluded to be quite suitable for dynamic analyses [13]. The different welfare dimensions have historically been classified into the following categories:

- *Health*. Health is important both as a part of human capital, and can compensate for or add to economic resources [9].
- *Housing*. Meaning access to housing and amenities.
- *Education*. Education can compensate for or add to economic resources.
- *Employment/occupation*. Full employment has been described as the key to equality of labour income.
- *Economic resources*. Economic resources is regarded as important because of its close relationship to need satisfaction.
- *Social relations*, as in the presence of family and lasting social relationships, which create a sense of security and can serve as an important resource.
- *Political resources*. This category has been described as the individual’s ability to protect their own legal interests [11], both those concerning their own rights under different authorities but also concerning political activities.
- *Recreation*, which has been defined as having access to culture and recreation.
- *Security of life and property*, including, for example, being a victim of criminal activity directed towards their own person or toward their material possessions.

These universal social concerns have been described as having been sprung from big life projects that all humans face [9]. A lack of resources within a single area has been viewed as an indicator of a problem in terms of welfare. However, when discussing the welfare dimensions in relation to especially vulnerable groups within society the areas are not ideal, as these groups are small and poorly represented in surveys [14]. More severe resource scarsity have not been investigated in the level of living surveys, and aspects like substance abuse may be extremely relevant for the recidivism of vulnerable groups [11]. In this paper, the assumption is made that aspects of importance for recidivism are also of importance for the overall life situation.

When applying the concept of welfare dimensions to vulnerable groups, the idea of what constitutes a ‘baseline’ occurrence for each different dimension needs to be adjusted. For example: if having any employment in the group is very rare, the presence of any employment will have to be treated as important and a differentiation from the norm. An area like ‘health’ for individuals with chronic serious mental disorders may need to be considered in terms of whether they are in need of inpatient care, rather than being free from health issues, and so on.

### Perspectives on welfare dimensions and reoffending

The importance of welfare dimensions when it comes to reoffending has been discussed from different perspectives, one of which is the resource perspective. From this perspective, a lack of resources can impact upon several important aspects of a person’s life; for example family conditions, financial resources, or health [15]. This, in turn, can have an effect on the individual’s propensity to commit a new crime, or to abstain. A lack of resources means that a person’s opportunities become more limited, which can explain a choice to commit new crime(s) [11]. Another theoretical perspective from which welfare dimensions can be interpreted is control theory [16]. Here, the assumption is that humans are prone to committing crimes and the reason why people generally do not is that social order requires us to conform to societal standards to be or remain successful. For individuals with problems in areas like social relations and fewer links to society to maintain, crime may thus be explainable [11]. This been empirically supported by research showing that offenders who lived with a spouse, parent, other relative, or in a residential program were less likely to recidivate than those who did not [17]. Prison inmates have also shown large differences compared to the rest of the population in level of living surveys [11].

While research on FFPs is lacking, knowledge about offenders without mental disorders may be generalisable to the situations of FFPs. Such existing research is largely qualitative, and has focused on what offenders themselves perceive to be of importance when abstaining from committing new crimes [18–21]. Research has shown that at the time of their release from prison, many offenders have severe problems of several kinds, the most important ones being practical issues like housing and money [20, 22]. Aspects like secured housing and clearly defined and effective communication pathways have also been highlighted as imperative for an individual’s transition from prison to the community [21, 23].

In addition to being convicted of a serious crime, FFPs have also all been diagnosed with a serious mental disorder. Having a mental disorder has been shown to be associated with an increased risk of not being employed and with not having secondary or higher education, leading to substantial loss of total earnings and income [24]. These findings, combined with what is known about offenders in general, suggest that FFPs will likely have accumulated problems in several welfare dimensions. This is distressing since an accumulation of several welfare problems points to a more vulnerable situation than problems in the different dimensions by themselves [11]. This accumulation further indicates the importance of investigating several different dimensions of an individual’s life situation together, rather than examining them as individual factors or dimensions by themselves. The different welfare dimensions together are needed to better understand the complexity of what an overall life situation looks like. Research on information known at the time of discharge has shown that aspects related to support and control were associated with a lower likelihood of post-discharge reconviction [6]. However, very little to no research has focused on the life situation of this vulnerable group. It is likely that factors influencing the likelihood of recidivism are also of importance to an individual’s overall level of living, but a wider investigation is needed.

### Forensic psychiatric care in Sweden

Every year, about 300 offenders in Sweden are sentenced to forensic psychiatric care [25] and about 115 individuals are discharged from forensic psychiatric care every year. The most common primary diagnosis is schizophrenia, and most (93%) had been in contact with psychiatric services at some point before their admission to forensic psychiatric care [26]. The median length of stay in forensic psychiatric services for discharged patients in Sweden is 58 months, or just under 5 years, which includes both in- and outpatient forensic psychiatric care [26]. Most patients receive mandated outpatient care for a period before they are formally discharged from all forensic psychiatric services.

Forensic psychiatric care is often combined with special court supervision (SCS). For patients receiving forensic psychiatric care with SCS (about 85%; Swedish National Forensic Psychiatric Register [26]), decisions about discharge are made by an administrative court. When the administrative court makes a decision about discharge, it considers both whether there is a risk of relapse into serious crime due to the offender’s mental disorder, and whether forensic psychiatric care is needed in consideration of the patient’s mental state or personal conditions [27]. This should mean that forensic psychiatric patients are discharged to well-ordered life situations, which would be important for the individual’s well-being but also for their risk of reoffending, since problems regarding professional services and plans, living situations, and personal support are risk factors for recidivism [28]. To help forensic psychiatric patients transition back into society, extensive planning must be in place at the time of discharge. Many Swedish forensic psychiatric facilities have therefore employed social workers [29], to work together with different welfare services regarding the patients need of housing and support at discharge and to ensure that the patient’s financial situations are in order.

Along with the other Nordic countries, Sweden has a comprehensive welfare system [30]. This fact makes it increasingly relevant to investigate how the life situation of different dimensions of welfare appear for this vulnerable group of individuals, applying a wider perspective than just studying the rates of criminal reconviction.

### Aim

This paper investigates the life situation for former forensic psychiatric patients, as reflected through several welfare indicators asking the following research questions:

- Are there different subgroups of FFPs regarding level of living post-discharge?
- Are there background factors are of importance for subgroup membership?

Using these research questions, the authors hope to gain more knowledge on what life looks like for this group. This will be done by empirical investigation of different configurations of the complex welfare dimensions, where the different configurations represent subgroups of typical life situations. The possible combinations of welfare resources and welfare problems will be viewed as outcomes in relation to both historical, clinical and situational factors.

## Methods

### Data and sample

The study was performed using retrospective pre-existing register data. The sample was acquired from the Swedish National Forensic Psychiatric Register (SNFPR) for the time-period 2009–2018. The SNFPR reported a 86% coverage rate of all forensic psychiatric patients in Sweden, with data from 24 out of 25 forensic psychiatric units in Sweden [31]. The SNFPR includes both pre-index crime background variables, variables concerning the time in forensic psychiatric care, and variables concerning the situation at the time for discharge from forensic psychiatric services. We constructed a database by combining the SNFPR data with data from the National Patient Register (NPR), the Longitudinal integrated database for health insurance and labour market studies (LISA), the Register of interventions according to the act concerning Support and Service for Persons with Certain Functional Impairments (the LSS register), and data on reconvictions from the National Council of Crime Prevention (NCCP).

Inclusion criteria were being included in the SNFPR and having been discharged from forensic psychiatric care between 2009 and 2018, excluding the patients whose forensic psychiatric care had ended because they had died. 1146 individuals matched the inclusion criteria, of which 938 (82%) were male, and 212 (18%) were female. Their average length of stay (combined time in both in- and outpatient forensic psychiatric care) was 4.9 years (*Md* = 3.7). Follow-up time varied from 4 to 3644 days, (*m* = 1697, *Md* = 1685). For 843 individuals (73%), the forensic psychiatric care was combined with SCS. Most (89%) had been sentenced to forensic psychiatric care due to a violent crime, and having a previous conviction (64%) and substance abuse (56%) was common. Mean age at discharge was 43.6 years (*Md* = 42).

### Welfare dimensions

The welfare dimensions used in the study were inspired by Swedish welfare research, but were adjusted to: (1) include only dimensions that apply to the post-discharge situation, and (2) include dimensions assumed to be relevant to FFPs overall life situation. Dimensions that are related to the risk of reoffending are assumed to also be relevant to the life situation as a whole.

Several of the original welfare dimensions used in level of living research were excluded. These were education (due to a low variation in the sample), recreation (due to a lack of register-based information), and security of life and property (due to a lack of register-based information). The remaining dimensions were operationalised to be included in the analyses. Two new dimensions were added: substance abuse and criminality. Substance abuse was included, as this has previously been acknowledged to be of importance to vulnerable groups [11], and criminality was included since prevention of reoffending is an objective for Swedish forensic psychiatric care.

#### Health

The FFPs had all been diagnosed with a serious mental disorder at the time of their forensic psychiatric investigation, which indicates that their psychiatric health is crucial to their overall well-being. The need for inpatient psychiatric care is a clear indicator of poor psychiatric health.

In this study the indicator of the dimension is having received psychiatric inpatient care post-discharge from forensic psychiatric care. (No/Yes), but below the median number of days (yes), or above the median number of days (no). Retrieved from the NPR.

#### Housing

Previous research has shown that having one’s main living accommodation being supported at the time of discharge is associated with a lower likelihood of reconviction [6], which makes it likely that this is a factor of importance for an individual’s level of living.

In this study, this dimension indicator is operationalised as whether the main living accommodation is supported with staff at the time of discharge and/or post-discharge. For a complete list of which variables are included, see Appendix I with information on the exact variables in Swedish. Retrieved from the SNFPR and the LSS register.

#### Substance abuse

An effective and available way for society to trace whether an individual has active substance abuse problems is whether this is recorded by health care services.

In this study the indicator of the dimension is operationalised as having received a substance abuse diagnosis in either in- or outpatient care post-discharge. Retrieved from the NPR.

#### Employment

Traditional employment is very uncommon in this group, and we have therefore widened the definition to include those who were assessed as being close to employment.

In this study the indicator of the dimension is operationalised as being registered as being in employment at any time post-discharge and/or receiving any of the welfare benefits connected to being close to employment. Retrieved from the LISA register.

#### Economic resources/security of income

The variance of total yearly income was low in the group. We posited that security of income, or rather insecurity of income, could be a stressor. Because of this, we divided the economic dimension into two different indicators; whether an individual has received any permanent welfare benefits (that are not re-evaluated regularly), and whether an individual has received any temporary welfare benefits (that are re-evaluated regularly and will eventually be withdrawn).

- Receives any permanent welfare benefit post-discharge (yes/no). Retrieved from the LISA register.
- Receives any temporary welfare benefit post-discharge (yes/no). Retrieved from the LISA register.

#### Social relations

Both presence and quality of social relations are difficult to measure using nationwide register data. However, spouses, live-in adult partners, and children are relationships on which information is available. This is also relevant, as research has shown that for offenders in general, living with someone is a factor relevant to understanding differences in recidivism [17].

In this study this is operationalised as having a registered partner and/or child(ren) that live with them or that they pay child support for. Retrieved from the LISA register.

#### Political resources

For FFPs, having a trustee or limited guardian is associated with a decrease in the likelihood of post-discharge recidivism [6]. These roles include helping the individual to protect their legal interests, which makes it a relevant indicator of political resources.

In this study the indicator of the dimension is operationalised as having a trustee or limited guardian (god man/förvaltare in Swedish) at the time of discharge from forensic psychiatric care and/or a facilitator (stödperson in Swedish) post-discharge. Retrieved from the SNFPR and the LSS register.

#### Criminality

In this study the indicator of the dimension is operationalised as a reconviction for any crime during the follow-up period. Retrieved from the NCCP.

For details about the prevalence of welfare indicators in the sample, see Table A1 in Appendix II.

### Background variables

We also wanted to investigate associations between class membership and different demographic/background variables. The included variables were chosen because they represent a mix of conventional demographic information (for example gender and age) and factors that previous research has shown to be of importance for recidivism rates (for example history of substance abuse and previous convictions), that can be retrieved for all FFPs. Included variables were:

- Gender (man/woman).
- Length of stay in forensic psychiatric care in years (continuous variable).
- Age at discharge in years (continuous variable).
- Born in Sweden (yes/no).
- Pre-index crime history of substance abuse (yes/no).
- Pre-index crime conviction (yes/no).
- Index crime (violent/non-violent): The following crimes were classified as violent including attempts to commit these crimes: homicide, manslaughter, assault and battery, arson, unlawful threats, violation of integrity, unlawful coercion, molestation, violence against an officer, robbery and sexual offenses including sexual molestation.
- Forensic psychiatric care with special court supervision (yes/no).
- Presence of any diagnosis of psychosis (yes/no), defined as any of the F20-F29 diagnoses according to International Statistical Classification of Diseases and Related Health Problems 10th Revision (ICD-10) as either primary or secondary diagnosis.
- Presence of any personality disorder (yes/no), defined as any of the F60-F69 diagnoses according to ICD-10 as either primary or secondary diagnosis.

Diagnostic information is presented in the broader categories of “any psychosis” or “any personality disorder”, to focus on the occurrence of different types of symptoms rather than specific diagnoses. This choice was also made because the number of individuals diagnosed with each of the different disorders within the category would have been too few to make statistical analyses meaningful. The choice of these specific groups was made both because of the relevance of these types of diagnoses to recidivism [32, 33] and because together they include the majority of this population.

### Analytical design

#### Are there different subgroups regarding level of living?

We wanted to identify subgroups of life situations, which is why we chose to perform latent class analysis (LCA) using the software Latent GOLD 6.0. LCA works from the assumption that membership in unobserved (latent) classes can be caused or explained by patterns of indicators. With this theoretical standpoint, scores or occurrences of indicator variables are viewed as driven by an individual’s class membership [34].

There is no clear common agreement on the best criteria for determining the number of latent classes in a model. The fit statistics are, of course, to be taken into consideration, but the use of a model with less good fit statistics can sometimes be justified if it is theoretically validated, or easier to interpret and therefore more meaningful to apply to classes [35]. Many different models, including a variety of different combinations of different welfare factors, were tested before deciding on the chosen model. Models typically improve when classes are added until the optimal solution is found, and then model quality decreases.

Classes were formed using the Bayesian Information Criterion (BIC), taking the likelihood ratio chi-squared statistic (L²), df, p-value and Entropy R² estimators into consideration. Lower BIC indicate better fit [34]. L² can be interpreted as indicating how much of the relationship between the variables remains unexplained by the model. A significant *p*-value indicates lack of model fit in absolute terms [36]. Entropy indicates how accurately a model defines classes [34]. As described by BE Weller, NK Bowen and SJ Faubert [34], we wanted a model where BIC was low, L² was not substantially larger than df, the p-value was larger than 0.05, and entropy was at least 0.8. It was also deemed necessary that the p-value for all indicators was less than 0.05, meaning that all indicators contributed significantly to discriminating between clusters [35].

#### Are there background factors of importance for subgroup membership?

To investigate which background variables were associated with class membership, we used multinominal logistic regression analysis. Multinominal logistic regression can be used to investigate the relationship between categorical and unordered dependent variables, which means that it can handle analyses with class membership as a dependent variables [37]. The estimated coefficients are presented as odds ratios relative to the reference category.

## Results

### Model selection

The main aim of the article was to investigate whether there are different subgroups regarding level of living, which was investigated by means of an LCA. During the process of model selection, multiple models were attempted. The models not matching the selection criteria of having both acceptable fit statistics and having all indicators contributing significantly to the model were discarded. Many different combinations and versions of theoretically-relevant indicators were tried and discarded before arriving at the final model, which was the model assessed as best describing the data.

Table 1 shows the 1–6 cluster versions of the final model. The analysis showed that the model with the best fit statistics is the 5 cluster version, which showed the lowest BIC and was close to the lowest AIC. When viewing the results, however, we decided to proceed by using the 4 cluster version on the same model. This was done because in the 5 cluster model, two of the classes were very similar and exhibited the same empirical implications, making the interpretation more difficult and less meaningful. In the 4 class model, the clusters were more distinct from each other, and was thus deemed to have better explanatory value. The importance of presenting a model with not only the best fit statistics but a meaningful interpretation has been highlighted by previous research [35].

**Table 1:** Indicators of fit for models with one through six latent classes

Classes	LL	BIC(LL)	AIC(LL)	L²	df	p-value	Entropy R²
1	-6634.1	13345.65	13290.17	2462.05	1135	0.00	1
2	-6221.8	12598.64	12487.67	1637.54	1124	0.00	0.71
3	-6034.0	12300.44	12133.99	1261.86	1113	0.00	0.73
4^b^	-5943.3	12196.60	11974.66	1080.54	1102	0.67	0.77
5^a^	-5851.4	12090.17	11812.75	896.63	1091	1	0.77
6	-5821.9	12108.76	11775.86	837.73	1080	1	0.77

^a^Best fitting model according to criterion

^b^Chosen model

### Characteristics of classes

**Fig. 1:**
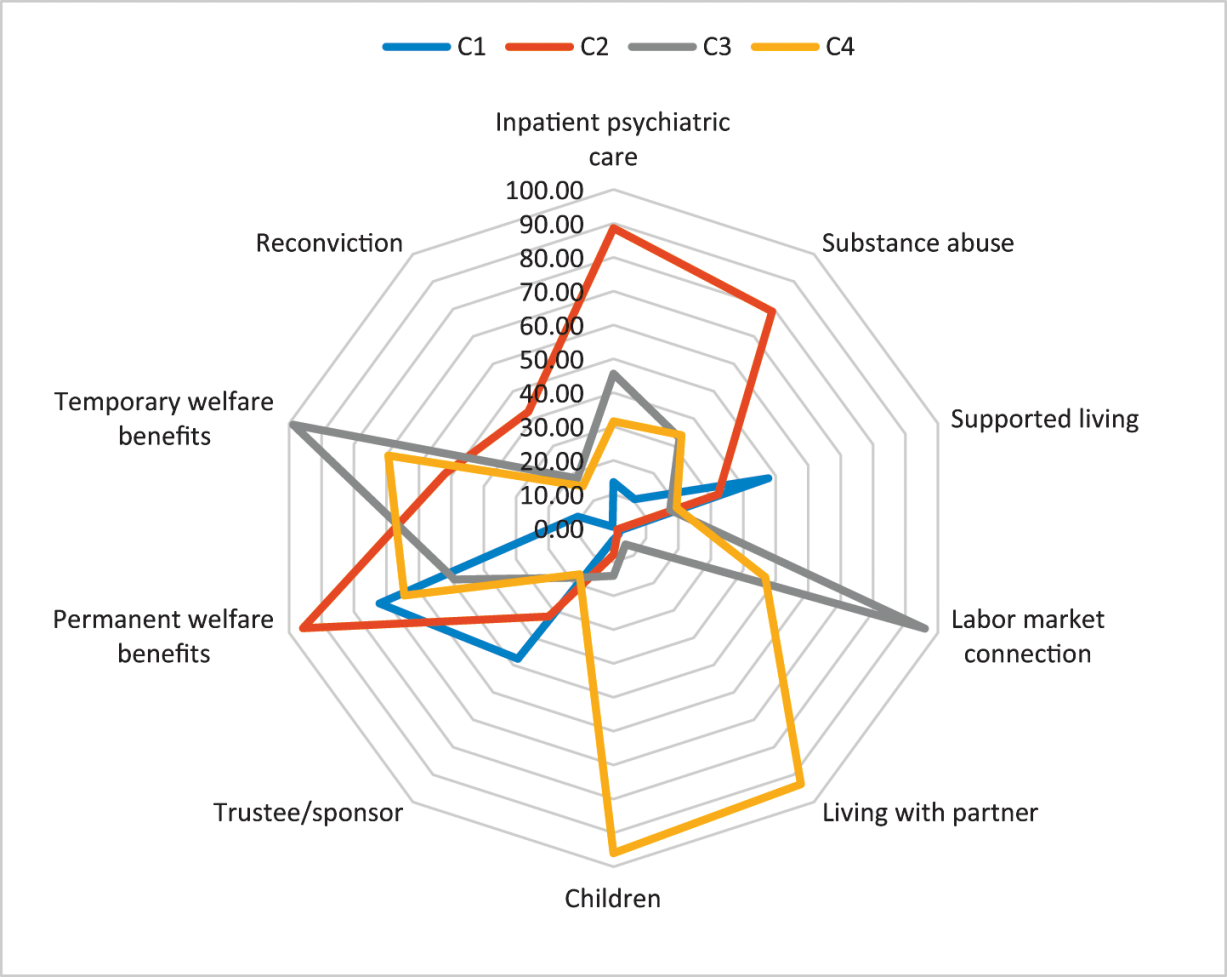
Latent class profiles of welfare dimensions post-discharge from forensic psychiatric care

After concluding that a 4 class model was best suited for describing the data, the characteristics of each class is described in Fig. 1. Figure 1 describes the resources of each identified latent class in each dimension of welfare, making it possible to compare the classes. For more details on the prevalence of each of the indicators in each class and in the total sample, see Table A1 in Appendix II.

#### Class 1: The high support group

Class 1 was the largest of the classes, representing 54% of the entire sample. This group was characterised by often having a supported living accommodation, formalised support, and receiving permanent welfare benefits. They rarely received inpatient psychiatric care, had low rates of substance abuse, and connections to the labour market. They seldom lived with a partner, had children, or received temporary welfare benefits, and were almost never reconvicted (1%).

#### Class 2: The general psychiatric needs group

Class 2 included 22% of the sample. This group was characterised by receiving high rates of psychiatric inpatient care, high rates of substance abuse, by often receiving both permanent and temporary welfare benefits, and were often reconvicted (43% of the group). These individuals seldomly had any connection to the labour market and seldom lived with a partner or had children.

#### Class 3: The working group

Class 3 comprised 13% of the sample. This group was characterised by having a very strong association with the labour market (96%) and most received temporary welfare benefits (99%). They seldom had a supported living accommodation, formalised support, or lived with a partner.

#### Class 4: The family group

Class 4 included 11% of the sample. This group was characterised by the fact that most of the group’s members lived with a partner (94%) and had children (96%). They also commonly had a connection to the labour market. It was equally common to receive temporary and permanent welfare benefits. They rarely received inpatient psychiatric care, had a supported living accommodation, or had formalised support.

### Class-specific probabilities

To give an idea about the similarities and differences between the classes we performed the Wald test for paired comparisons for each of the indicators (Table 2). As can be seen in Table 2, there are significant differences between the classes in the occurrence of all welfare dimension indicators. The *high support* group is more likely than all other groups to have a supported living accommodation, have formalised support, and receive permanent welfare benefits, and they are also more likely to have active substance abuse than both the *working group* and the *family group*. The *general psychiatric needs group* is more likely than all other groups to have active substance abuse, to receive inpatient psychiatric care, and to be reconvicted. They are also more likely than both the *working* and *family* groups to have a supported living accommodation, to have formalised support, and to receive permanent welfare benefits. The *working group* was more likely than all other groups to have a labour market connection and to receive temporary welfare benefits. The *family group* was more likely than all other groups to live with a partner and to have at least one child. They were also more likely than the *high support group* and the *general psychiatric needs group* to have a connection to the labour market.

**Table 2:** Class-specific probabilities/means of post-discharge welfare areas using Wald test for paired comparisons

	Class 1 (*N* = 619) P	Class 2 (*N* = 254) P	Class 3 (*N* = 149) P	Class 4 (*N* = 124) P	Wald test for paired comparisons
Substance abuse	0.20	0.53	0.15	0.12	2 > 1, 3, 4; 1 > 3, 4
Supported living	0.68	0.21	0.06	0.06	1 > 2, 3, 4; 2 > 3, 4
Labour market connection	0.07	0.03	0.61	0.29	3 > 1, 2, 4; 4 > 1, 2
Living with partner	0.10	0.05	0.06	0.78	4 > 1, 2, 3
Has children	0.13	0.11	0.11	0.65	4 > 1, 2, 3
Formalized support	0.69	0.20	0.06	0.05	1 > 2, 3, 4; 2 > 3, 4
Permanent welfare benefits	0.52	0.29	0.10	0.10	1 > 2, 3, 4; 2 > 3, 4
Temporary welfare benefits	0.15	0.29	0.36	0.20	3 > 1, 2, 4; 2 > 1, 4; 4 > 1
Reconvicted within follow-up time	0.04	0.64	0.20	0.12	2 > 1, 3, 4; 3, 4 > 1
Inpatient psychiatric care					2 > 1, 3, 4
No	0.70	0.05	0.13	0.12	
Below median	0.34	0.36	0.18	0.12	
Above median	0.09	0.66	0.16	0.08	

### Background variables and class membership

We have seen that the level of living of the FFPs can be understood in terms of four different groups with different resources in the different welfare dimensions, and will now investigate which background variables are associated with group membership.

Table 3 shows a multinominal logistic regression analysis of background variables for the classes as compared to the *high support group*, which was chosen because it is the largest of the classes and therefore assumed to be the ‘typical’ case of post-discharge life situation. Compared to the *high support group*, the *general psychiatric needs group* was shown to be younger (OR = 0.98, *p* > .00) and have a shorter length of stay in forensic psychiatric services (OR = 0.93, *p* > .00), while also being more likely to have pre-index crime substance abuse (OR = 3.21, *p* > .00) and to have at least one pre-index crime conviction (OR = 2.39, *p* > .00). The *working group* was shown to be younger (OR = 0.95, *p* > .00) and have a shorter length of stay (OR = 0.81, *p* > .00) in forensic psychiatric services. The *family group* is shown to be younger (OR = 0.93, *p* > .00) and have a shorter length of stay (OR = 0.88, *p* > .00) in forensic psychiatric services. They were also less likely to have been born in Sweden (OR = 0.53, *p* > .00), and more likely to have a personality disorder (OR = 1.70, *p* > .05).

**Table 3:** Multinominal logistic regression analysis comparing background variables between classes

Class^a^	Background variable	Sig.	OR	[95% CI]
Class 2	Age at discharge (years)	**	0.98	[0.97, 0.99]
	Length of stay (years)	**	0.93	[0.89, 0.97]
	Male sex (ref. = female)	0.83	1.05	[0.68, 1.61]
	Pre-index crime substance abuse	**	3.21	[2.20, 4.70]
	Pre-index crime conviction(s)	**	2.39	[1.60, 3.58]
	Violent index crime	0.20	0.72	[0.44, 1.19]
	Special court supervision	0.93	0.88	[0.05, 14.88]
	Born in Sweden	0.78	0.95	[0.66, 1.36]
	Presence of any psychosis	0.18	1.27	[0.90, 1.80]
	Presence of any personality disorder	0.18	1.33	[0.87, 2.03]
Class 3	Age at discharge (years)	**	0.95	[0.94, 0.97]
	Length of stay (years)	**	0.81	[0.75, 0.88]
	Male sex (ref. = female)	0.49	0.84	[0.52, 1.37]
	Pre-index crime substance abuse	0.55	0.88	[0.58, 1.33]
	Pre-index crime conviction	0.18	1.34	[0.87, 2.06]
	Violent index crime	0.11	0.61	[0.33, 1.12]
	Special court supervision	0.09	1.54	[0.94, 2.53]
	Born in Sweden	0.33	0.81	[0.54, 1.23]
	Presence of any psychosis	0.15	0.74	[0.50, 1.12]
	Presence of any personality disorder	0.75	0.92	[0.53, 1.57]
Class 4	Age at discharge (years)	**	0.93	[0.91, 0.95]
	Length of stay (years)	**	0.88	[0.82, 0.95]
	Male sex (ref. = female)	0.59	1.17	[0.66, 2.07]
	Pre-index crime substance abuse	0.34	1.25	[0.79, 1.97]
	Pre-index crime conviction	0.43	1.21	[0.76, 1.94]
	Violent index crime	0.23	0.67	[0.34, 1.30]
	Special court supervision	0.32	1.32	[0.76, 2.29]
	Born in Sweden	**	0.53	[0.34, 0.83]
	Presence of any psychosis	0.09	0.68	[0.43, 1.06]
	Presence of any personality disorder	*	1.70	[1.01, 2.86]

*p < .05, **p < .001

*Note*: ^a^The reference class is Class 1

## Discussion

In this study, we performed a latent class analysis of the post-discharge level of living for FFPs, using indicators representing different welfare dimensions. Furthermore, we performed multinominal logistic regression analysis in order to investigate potential associations between background variables and subgroup membership. We found that there are four classes representing typical patterns of level of living, and that these classes differ significantly regarding level of living. We also found that there are background factors that are associated with subgroup membership, including age at discharge, length of stay in forensic psychiatric care, and pre-index crime substance abuse and criminal conviction(s).

It is not up to us as authors of this article to pass judgment on what is a good or desirable life situation, and generally, we will take a neutral stance on what welfare indicators occur in these different life situations. However, there are welfare indicators that will be discussed and referred to as adverse outcomes. These include needing inpatient psychiatric care, having active substance abuse problems, and being reconvicted in a criminal court.

What is perhaps to be considered the primary result of the LCA is that the largest subgroup (the *high support group*) consists of more than half the population. This group is characterised by receiving high amounts of societal support in the form of permanent welfare benefits, supported living accommodation, and often having a trustee/limited guardian/facilitator—but also by remarkably low rates of adverse outcomes. This group seldom receives inpatient care, seldom has active substance abuse, and are almost never reconvicted. Group membership was associated with higher age at discharge and longer forensic psychiatric length of stay. It can be argued that because the subgroup is so large, the level of living of the individuals belonging to this subgroup is the typical level of living for a Swedish FFP post-discharge. This would reflect well on Swedish forensic psychiatric care, as well as on the administrative courts and the system deciding when to discharge forensic psychiatric patients, and on the Swedish welfare system given how these individuals are taken care of by society. Potentially, the low rates of adverse outcomes can be attributed to the extensive amount of societal support provided to these individuals. Of course, it cannot be ruled out that FFPs would do well in any system, as the results of this study do not answer any questions about causality. However, it is still encouraging that there is a large subgroup with few adverse outcomes. At the same time, it is worth remembering that the results here do not reflect subjective well-being.

The second largest group, the *general psychiatric needs group*, is characterised by the occurrence of several adverse welfare indicators: they often receive inpatient psychiatric care, have an active substance abuse, and have a high rate of reconviction. The 40% reconviction rate of this group is near that of former prisoners [see 38 for a comprehensive review], and 70% of those who were reconvicted belonged to this subgroup. As membership in this group was associated with having a pre-index crime history of both substance abuse and previous conviction(s), this is in line with previous research for both FFPs and offenders in general. ASince both factors are among the most important risk factors for crime [39–41], the high recidivism rates of this group may be explained by previous criminality and both previous and current substance abuse, rather than by other welfare dimensions.

The fact that there are two groups—the *working group* and the *family group*—for which group membership is associated with shorter length of stay, while the post-discharge situation includes less societal support and is still *not* associated with the different adverse outcomes, is of course good news. Identifying these individuals pre-discharge is important, and something that the existing system seems to have managed to do well. This, too, signals that the system works well as far as identifying which patients need support post-discharge and which patients do not.

The finding that the *family group* showed low rates of reconviction despite more often having a personality disorder is also interesting. Previous research has shown an increased risk for repeat offending in offenders with a personality disorder [32]. Our results suggest that the presence of close family, and perhaps the social control that this entails, mediates the effects of personality disorder on recidivism. However, there are also alternative interpretations of these results. For example, it is possible that crimes committed by members of this group are not perpetrated mainly towards stranger victims, which is generally the most common for FFPs [42], but toward family members. This may make it less likely for crimes that occur to be prosecuted and lead to a conviction.

All four subgroups demonstrate a high frequency of permanent welfare benefits. It is likely that this in part stems from debilitating effects of psychosis, and the passivation that comes from spending years in closed institutional settings. Both these factors can also be reflected in the overall absence of traditional employment. The whole sample demonstrates very low rates of labour market connection, with only 19% of the whole sample having any connection to the labour market at any time during the follow-up period. Since the definition we apply is exceptionally wide, including not only being in employment but also receiving welfare benefits associated with being close to being employed, the actual employment rate is even lower than 19%. This is not surprising, as previous research has shown that all serious mental disorders is associated with unemployment [24].

The low occurrence of certain dimensions raises questions about the relevance of applying welfare dimensions constructed for the general population on groups that (like this one) live under so drastically different conditions. It can be argued that the welfare dimensions are well-suited to describing the vulnerability of FFPs and their differentiation from the rest of the population. However, as the FFPs instead score higher on dimensions that are uncommon for the populations at large (for example substance abuse and reconviction), the welfare dimensions in their original form are not enough to describe the level of living of this group. This has also been previously discussed [11]. At the very least, the results of this study support the use of modified welfare dimensions for groups as vulnerable as this one.

## Limitations

Naturally, there are limitations to this study. One is a difficulty that is shared by all research utilising pre-existing register data, which is the fact that item definitions are limited to the definitions used by the register. For example, the variable “living with partner” only includes what the LISA register could possibly collect from official records. This includes whether or not someone shares a living accommodation with someone to whom they are married or registered as a partner, or with whom they have a child. Other constellations, such as having a partner but not cohabitating, or cohabitating without having children or being married, will not be caught by this register’s definition. It would also have been valuable to include more information on social network and whether there is any meaningful contact with relatives other than a partner. However, this kind of information does not exist in any population-based registers, and could therefore not be included.

The definitions of occurrence of indicators are also unrefined, and will in some cases only reflect specific moments in time. For example, having a connection to the labour market for a very short time during the follow-up period and then having no connection to the labour market at all for most of the time will have been coded as having a connection to the labour market, which may provide a somewhat inaccurate description of the bigger picture. This must be considered when interpreting the results. However, the size of the sample and the overall quality of registers, combined with the large amount of utilised information, means that the overall picture of FFP’s level of living is quite meaningful and valid. This gives unique insight into the lives of this group.

## Conclusion and future research

The fact that there are clear subgroups regarding level of living post-discharge, and that they also differ from each other regarding background variables, can be used by practitioners. Individual assessments will always be needed, but knowledge about these subgroups could be drawn upon to make informed decisions about in- and outpatient forensic psychiatric care, discharge from forensic psychiatric services, and what support is offered to FFPs.

The fact that the largest subgroup demonstrates so few adverse outcomes is very positive. Even though the design of this study does not support causal claims as to why this group does so well post-discharge, it is likely that the support by which the *high support* group is characterised by is connected to their success. It has previously been suggested that the risk of recidivism in FFPs is strongly related to the level of supervision [39], which is supported by the fact that factors associated with support at the time of discharge from forensic psychiatric care are associated with being less likely to be reconvicted post-discharge [6]. Supervision and support share several characteristics, and both support and the human connections that support entail can also be interpreted as a form of social control. For FFPs, who seldom live with a partner or have children, the formalised support offered by society may be especially urgent.

Future research could favourably include even more detailed information on the living situation and social relations of the group. It would also be interesting to study the trajectories of the lives of FFPs, something this method of study did not allow. To be able to utilise the results of this study in practice, it would also be beneficial to further investigate what situational factors are associated with different life situations; for example, length of stay in in- and outpatient care. Another important addition to this type of research would be investigating the subjective experiences FFPs have regarding their level of living. This would help both forensic psychiatric services, social services and other authorities involved in support to better understand what can be done to prevent life situations where these vulnerable individuals end up lacking key resources in welfare dimensions.

### Electronic supplementary material

Below is the link to the electronic supplementary material.

Supplementary Material 1: Appendix I

Supplementary Material 2: Appendix II

## Data Availability

Data are available from the registers that are included in the study. Since the registers are not publicly available, restrictions regarding the availability of the data apply. However, upon reasonable request and with permission of the included registers data are available from the authors. Please contact corresponding author at ebba.noland@umu.se for further questions.

## Abbreviations

FFP: Former forensic psychiatric patient
ICD-10: International Statistical Classification of Diseases and Related Health Problems 10th Revision
LCA: Latent class analysis
LISA: Longitudinal integrated database for health insurance and labour market studies
The LSS register: the Register of interventions according to the act concerning Support and Service for Persons with Certain Functional Impairments
MDO: Mentally disordered offender
NCCP: National Council of Crime Prevention
NPR: National Patient Register
SCS: Special court supervision
SNFPR: Swedish National Forensic Psychiatric Register
