# Supplementary material for: What happens after forensic psychiatric care? A latent class analysis of dimensions of welfare for former forensic psychiatric patients
Source: BMC Psychiatry. 2023 Dec 12;23:937. doi: 10.1186/s12888-023-05428-x (PMC10714544; doi:10.1186/s12888-023-05428-x)
Supplement: Supplementary file 1 — Supplementary Material 1: Appendix I [file 12888_2023_5428_MOESM1_ESM.docx]

Appendix I

**Housing**

- Bostad med särskild service för vuxna/annan särskilt anpassad bostad för vuxna (LSS)

**Employment**

- Studiemedel/studiehjälp
- Sjuk/arbetsskadeersättning
- Rehabiliteringsersättning
- Ersättning i samband med arbetsmarknadspolitisk åtgärd

**Permanent welfare benefit**

- Handikappersättning
- Ålders-/tjänstepension
- Förtidspension/sjukbidrag

**Temporary welfare benefit**

- Sjuk-/arbetsskadeersättning
- Socialbidrag
- Arbetslöshetsersättning
- Rehabiliteringsersättning
- Ersättning i samband med arbetsmarknadspolitisk åtgärd
