# Supplementary material for: What happens after forensic psychiatric care? A latent class analysis of dimensions of welfare for former forensic psychiatric patients
Source: BMC Psychiatry. 2023 Dec 12;23:937. doi: 10.1186/s12888-023-05428-x (PMC10714544; doi:10.1186/s12888-023-05428-x)
Supplement: Supplementary file 2 — Supplementary Material 2: Appendix II [file 12888_2023_5428_MOESM2_ESM.docx]

Appendix II

**Table A1.** Descriptive variables per class and for the total sample (*N* = 1146).

| Variable | Total sample (*N* = 1146)  N (%) | Class 1 (*N* = 619)  N (%) | Class 2 (*N* = 254)  N (%) | Class 3 (*N* = 149)  N (%) | Class 4 (*N* = 124)  N (%) | Wald | *p* |
| --- | --- | --- | --- | --- | --- | --- | --- |
| **Post-discharge** |  |  |  |  |  |  |  |
| Inpatient psychiatric care |  |  |  |  |  | 95.83 | ** |
| No | 730 (64) | 535 (86) | 29 (11) | 81 (54) | 85 (69) |  |  |
| Below median | 203 (18) | 62 (10) | 85 (34) | 34 (23) | 22 (18) |  |  |
| Above median | 213 (19) | 22 (4) | 140 (55) | 34 (23) | 17 (14) |  |  |
| Substance abuse | 357 (31) | 65 (11) | 201 (79) | 49 (33) | 42 (34) | 30.16 | ** |
| Supported living | 427 (37) | 295 (48) | 82 (32) | 26 (17) | 24 (19) | 97.97 | ** |
| Labour market connection | 217 (19) | 13 (2) | 2 (1) | 143 (96) | 58 (47) | 27.48 | ** |
| Living with partner | 145 (13) | 15 (2) | 5 (2) | 9 (6) | 116 (94) | 24.38 | ** |
| Has children | 181 (16) | 21 (3) | 20 (8) | 21 (14) | 119 (96) | 18.96 | ** |
| Formalized support | 425 (37) | 295 (48) | 82 (32) | 27 (18) | 21 (17) | 102.73 | ** |
| Permanent welfare benefits | 842 (73) | 446 (72) | 243 (96) | 73 (49) | 80 (65) | 72.35 | ** |
| Temporary welfare benefits | 433 (38) | 68 (11) | 132 (52) | 147 (99) | 86 (69) | 4.44 | * |
| Reconvicted within follow-up time | 157 (14) | 3 (1) | 108 (43) | 27 (18) | 19 (15) | 93.74 | ** |
| **Background/demographic variables** |  |  |  |  |  | Chi-square | *p* |
| Sex |  |  |  |  |  | 1.06 | .79 |
| Men | 938 (82) | 500 (81) | 213 (84) | 119 (80) | 104 (84) |  |  |
| Women | 212 (18) | 119 (19) | 41 (16) | 30 (20) | 20 (16) |  |  |
| Pre-index crime conviction | 738 (64) | 362 (59) | 210 (83) | 89 (60) | 75 (61) | 19.48 | ** |
| Pre-index crime substance abuse | 646 (56) | 306 (49) | 202 (80) | 68 (46) | 68 (55) | 45.92 | ** |
| Violent index crime | 1021 (89) | 569 (92) | 215 (85) | 128 (86) | 107 (86) | 3.41 | .33 |
| Special court supervision | 843 (73) | 466 (75) | 169 (67) | 111 (75) | 95 (77) | 7.70 | * |
| Born in Sweden | 783 (68) | 432 (70) | 182 (72) | 95 (64) | 71 (57) | 8.01 | * |
| Presence of any psychosis | 685 (60) | 381 (62) | 166 (65) | 78 (52) | 59 (48) | 8.47 | * |
| Presence of any personality disorder | 208 (18) | 97 (16) | 53 (21) | 24 (16) | 32 (26) | 5.65 | .13 |
|  | *M* (Md) | *M* (Md) | *M* (Md) | *M* (Md) | *M* (Md) | Chi-square | *p* |
| Age (in years) at discharge | 43.6 (42) | 47 (46) | 42.7 (42.5) | 38.2 (37) | 35.7 (33) | 90.89 | ** |
| Length of stay (combined time in in- and outpatient care) | 4.9 (3.7) | 5.7 (4.4) | 4.5 (3.4) | 3.3 (2.6) | 3.7 (3) | 48.72 | ** |

*p < .05, **p < .001
